# Riboflavin and pyridoxine restore dopamine levels and reduce oxidative stress in brain of rats

**DOI:** 10.1186/s12868-018-0474-4

**Published:** 2018-11-09

**Authors:** Armando Valenzuela Peraza, David Calderón Guzmán, Norma Osnaya Brizuela, Maribel Ortiz Herrera, Hugo Juárez Olguín, Miroslava Lindoro Silva, Belén Juárez Tapia, Gerardo Barragán Mejía

**Affiliations:** 1 Laboratorio de Neurociencias, Instituto Nacional de Pediatría (INP), Mexico City, Mexico; 2 Laboratorio de Bacteriología Experimental, INP, Mexico City, Mexico; 3 Laboratorio de Farmacología, Instituto Nacional de Pediatría (INP), y Facultad de Medicina, Universidad Nacional Autónoma de México, Av Imán #1, 3er piso, Col Cuicuilco, CP 04530 Mexico City, Mexico

**Keywords:** Brain, Huntington animal model, Oxidative stress, Riboflavin, Pyridoxine

## Abstract

**Background:**

Neurological disorders suggest that the excitotoxicity involves a drastic increase in intracellular Ca^2+^ concentrations and the formation of reactive oxygen species. The presence of these free radicals may also affect the dopaminergic system. The aim of this work was to determine if riboflavin (B_2_) and pyridoxine (B_6_) provide protection to the brain against free radicals generated by 3-nitropropionic acid (3-NPA) by measuring the levels of dopamine (DA) and selected oxidative stress markers.

**Methods:**

Male Fisher rats were grouped (n = 6) and treated as follows: group 1, control (NaCl 0.9%); group 2, 3-NPA (20 mg/kg); group 3, B_2_ (10 mg/kg); group 4, B_2_ (10 mg/kg) + 3-NPA (20 mg/kg); group 5, B_6_ (10 mg/kg) and group 6, B_6_ + 3-NPA. All treatments were administered every 24 h for 5 days by intraperitoneal route. After sacrifice, the brain was obtained to measure DA, GSH, and lipid peroxidation, Ca^2+^, Mg^2+^, ATPase and H_2_O_2_.

**Main findings:**

Levels of dopamine increased in cortex, striatum and cerebellum/medulla oblongata of animals that received 3-NPA alone. The lipid peroxidation increased in cortex, striatum, and cerebellum/medulla oblongata, of animals treated with B_2_ vitamin alone. ATPase dependent on Ca^+2^, Mg^+2^ and H_2_O_2_ increased in all regions of animals that received 3-NPA alone.

**Conclusion:**

The results confirm the capacity of 3-NPA to generate oxidative stress. Besides, the study suggests that B_2_ or B_6_ vitamins restored the levels of DA and reduced oxidative stress in brain of rats. We believe that these results would help in the study of neurodegenerative diseases.

## Background

The main incident in neurological disorders is excitotoxicity which entails an extensive upsurge in the concentrations of intracellular Ca^2+^ and the production of reactive species such as ROS and RNS by lethal pathways [1]. In addition, there are alterations of the mitochondrial ultrastructure and DNA damage caused by nitric oxide- (NO-) dependent oxidative stress [2]. This damage is the primary event in 3-nitropropionic acid (3-NPA) toxicity.

In Wistar rats, it has been reported that 3-nitropropionic acid leads to neurodegeneration and that the intravenous administration in rats provides valuable insight of Huntington’s disease model [3]. There is evidence that metabolism of transmitter dopamine by monoamine oxidase enzyme may promote striatal damage in mitochondrial toxin induced models of Huntington’s disease (HD) [4], and that HD is a devastating neurodegenerative disorder that reflect neuronal dysfunction and ultimately death in selected brain regions with striatum and cerebral cortex being the principal targets [5]. Some nutrients are known to act as antioxidants; and although neglected as an antioxidant, riboflavin is one of such nutrients that independently or as a component of the glutathione redox cycle has an important antioxidant action [6]. On the other hand, pyridoxine alters serotonin metabolism [7]. Both compounds are water-soluble vitamins that possess antioxidant activity [8].

Neuromodulation activity of the NO- is a documented fact, however, an excess amount of it can produce oxidative damage or nitroso-glutathione (NOGSH) inside the cell, thus; causing the damage of the cell [9]. It is known that free radicals (FR) induce damage to the components of the cell [10]. Such FR-induced damage particularly affects the plasma membrane lipids [11]. In addition, the cells of the central nervous system are extremely susceptible to these unpaired electron molecular species. Nitric oxide (NO) acts on hypothalamic neurocircuits and on higher brain circuits, e.g. dopaminergic system to regulate energy and glucose homeostasis [12]. Therefore, the presence of FR may upset this regulatory function of NO. The structural proteins in the lipid bilayer are contiguous with brain plasma membrane phospholipids [13] and the ionic inflow and outflow through the lipid bilayer is maintained by Na^+^, K^+^ ATPase enzyme which stimulates Na^+^ and K^+^ flows [14]. It is know that when the activity of the enzyme Na^+^, K^+^ ATPase is inhibited, it triggers the release of excitatory amino acid in the CNS [15].

In view of all the aforementioned, the objective of the present work was to make a comparative analysis of the protective effects derivable from riboflavin (B_2_) and pyridoxine (B_6_) on 5-HIAA and dopamine levels, as well as on selected markers of oxidative stress in rats’ brain regions after an induction of Huntington’s disease (HD). Literature reports suggest that these substances may participate in the neutralization of excess free radicals in oxidation mechanisms. The production of free radicals, a usual biological phenomenon, is regulated by different metabolic routes, which constitute the first line of defence in the human body.

The aim of this work was to determine if riboflavin (B_2_) and pyridoxine (B_6_) provide protection to the brain against free radicals generated by 3-nitropropionic acid (3-NPA) by measuring the levels of dopamine (DA) and selected oxidative stress markers.

## Materials and methods

Thirty-six male Wistar rats (250 g) were procured from Bioterium of Metropolitan University of Mexico City and housed in clean plastic cages, separated into 6 groups and treated as follows: group 1, control (NaCl 0.9%); group 2, 3-NPA (20 mg/kg); group 3, B_2_ (10 mg/kg); group 4, B_2_ (10 mg/kg) + 3-NPA (20 mg/kg); group 5, B_6_ (10 mg/kg); group 6, B_6_ (10 mg/kg) + 3-NPA (20 mg/kg), each group N = 6. The administration of treatments was by i.p. The animals received the drugs every 24 h during 3 days. At the end of the treatment period and 30 min after the last drug administration, the animals were sacrificed with guillotine without anaesthetic procedure. The animal brains were immediately extirpated and put in saline solution (NaCl 0.9%) at 4 °C. Tissues were immediately dissected in regions and used to evaluate reduced glutathione (GSH), H_2_O_2_, lipid peroxidation, ATPase, and the levels of DA and 5-hydroxyindolacetic acid (5-HIAA).

The rats or breeds employed in the study were subjected to a selection process based on phenotypic variety; genetic, environmental and compartmental factors. Longitudinal weight curves, weight and physical exploration were the means employed to select a breed for inclusion or exclusion in the study. Also, to select a breed, it is fundamental that inbreeding is non-existence, thus having a hereditary control of traits with continuous variation.

The selected animals were kept in cool and dry place at a temperature of 15–16 °C and with air filter and humidity of between 50 and 60. The place was maintained clean and was continuously sterilized to avoid bacterial and fungal growth.

The breeds were fed with standardized diet based on 3800 kcal/kg, proteins 12%, fat 5%, vitamins and minerals. The selected animals were 3 months old male Wistar of approximately 250 g weight.

### Brain extraction

On sacrificing the animals, the brains were excised from the base. Then, the brain tissue was dissected in cortex, striatum and cerebellum/medulla oblongata, weighed and homogenised in 5 volumes of 0.05 M tris–HCl, pH 7.4. An aliquot of the homogenized brain tissue was obtained and again homogenised in 0.1 M perchloric acid (HClO_4_) (50:50 v/v) using Yamato homogenizer (Yamato lh-21 LSC Lab, Dallas, USA) and stored at − 20 °C until analysed.

Animal management and care was conducted in accordance to the international guidelines for animal care and to the Mexican Guidelines ZOO-062. Besides, the study was approved by the Laboratory Animals Use and Care Committee of National Institute of Pediatrics.

### Measurement of Dopamine (DA)

The DA levels were measured in the supernatant of tissue homogenized in HClO4 after centrifugation at 5000*g* for 10 min in a microcentrifuge (Hettich Zentrifugen, model Mikro 12-42, Tuttlingen, Germany), with a version of the technique reported by Calderon et al. [16]. An aliquot of the HClO4 supernatant, and 1.9 mL of buffer (0.003 M octyl-sulphate, 0.035 M KH_2_PO_4_, 0.03 M citric acid, 0.001 M ascorbic acid), were placed in a test tube. The mixture was incubated for 5 min at room temperature in total darkness, and subsequently, the samples were read in a spectrofluorometer (Perkin Elmer LS 55, Buckinghamshire, England) with 282 nm excitation and 315 nm emission lengths. The FL Win Lab version 4.00.02 software was used. Values were inferred in a previously standardized curve and reported as nMoles/g of wet tissue.

### Measurement of 5-HIAA

5-HIAA levels were measured in the supernatant of tissue homogenized in HClO_4_ after centrifugation at 5000*g* for 10 min in a microcentrifuge (Hettich Zentrifugen), with a modified version of the technique reported by Beck et al. [17]. An aliquot of the HClO_4_ supernatant, and 1.9 mL of acetate buffer 0.01 M pH 5.5 were placed in a test tube. The mixture was incubated for 5 min at room temperature in total darkness, and subsequently, the samples were read in a spectrofluorometer (Perkin Elmer LS 55) with 296 nm excitation and 333 nm emission lengths. The FL Win Lab version 4.00.02 software was used. Values were inferred in a previously standardized curve and reported as nMoles/g of wet tissue.

### Measurement of reduced glutathione (GSH)

GSH levels were measured from the supernatant of the perchloric acid homogenised tissue, obtained after centrifuging at 5000*g* for 5 min (Hettich Zentrifugen) according to a modified method of Hissin and Hilf [18]. A 1.8 mL phosphate buffer pH 8.0 with EDTA 0.2% plus a 20 μL aliquot of the supernatant and 100 mL of ortho-phthaldehyde (OPT) 1 mg/mL in methanol were put in a test tube and mixed. The mixture was then incubated for 15 min at room temperature in absolute darkness. At the end of the incubation time, the samples were read in a spectrophotometer (Perkin Elmer LS 55), with excitation and emission wavelengths of 350 and 420, respectively. FL Win Lab version 4.00.02 software was used. Values were inferred from a previously standardised curve and expressed as nM/g.

### Measurement of lipid peroxidation

The lipid peroxidation across the reactive substances to the thiobarbituric acid (Tbars) determination was carried out using the modified technique of Gutteridge and Halliwell [11], as described below: From the homogenized brain in tris–HCl 0.05 M pH 7.4, 1 mL was taken and to it was added 2 mL of thiobarbituric acid (Tba) containing 1.25 g of Tba, 40 g of trichloroacetic acid (Tca), and 6.25 mL of concentrated chlorhydric acid (HCl) diluted in 250 mL of deionized H_2_O. The mixture was heated to boiling point for 30 min. (Thermomix 1420) and then cooled in an ice bath for 5 min. after which it was centrifuged at 700*g* for 15 min. (Sorvall RC-5B Dupont). The absorbance of the floating tissues was read in triplicate at 532 nm in a spectrophotometer (Helios de UNICAM). The concentration of TBARS was expressed in µM of Malondialdehyde/g of wet tissue.

### Measurement of total ATPase

The activity of ATPase was assayed according to the method proposed by Calderón et al. [19]. 1 mg (10%) w/v of homogenised brain and heart tissues in tris–HCl 0.05 M pH 7.4 was incubated for 15 min in a solution containing 3 mM MgCl_2_, 7 mM KCl, and 100 mM NaCl. To this was added 4 mM tris-ATP and incubated for another 30 min at 37 °C in a shaking water bath (Dubnoff Labconco, TX, USA). 100 µL trichloroacetic acid at 10% w/v was used to stop the reaction and the samples were centrifuged at 100*g* for 5 min at 4 °C. Inorganic phosphate (Pi) was measured in triplicates using one supernatant aliquot as proposed by Fiske and Subarrow [20]. Supernatant absorbance was read at 660 nm in a Helios-α, UNICAM spectrophotometer and expressed as mM Pi/g wet tissue per minute.

### Measurement of H_2_O_2_

The determination of H_2_O_2_ was made using the modified technique of Asru [21]. Each brain region (cortex, striatum, cerebellum/medulla oblongata) was homogenized in 3 mL of tris–HCl 0.05 M pH 7.4 buffers. From the diluted homogenates, 100 µL was taken and mixed with 1 mL of potassium dichromate solution (K_2_Cr_2_O_7_). The mixtures were heat to boiling point for 15 min (Thermomix 1420, CA, USA). The samples were later placed in an ice bath for 5 min and centrifuged at 3000*g* for 5 min (Hettich Zentrifugen). The absorbance of the floating was read in triplicate at 570 nm in a spectrophotometer (Heλios-α of UNICAM, Bristol, UK). The concentration of H_2_O_2_ was expressed in µMoles.

### Statistical analysis

One way analysis of variance (ANOVA) or Non parametric Kruskal–Wallis test was used after the data have been subjected to variances homogeneity test. Post hoc Tukey–Kramer or Steel–Dwass contrast was employed. The values of *p* < 0.05 were considered statistically significant [22]. JMP Statistical Discovery Software version 10.0 from SAS was used.

## Results

**Fig. 1:**
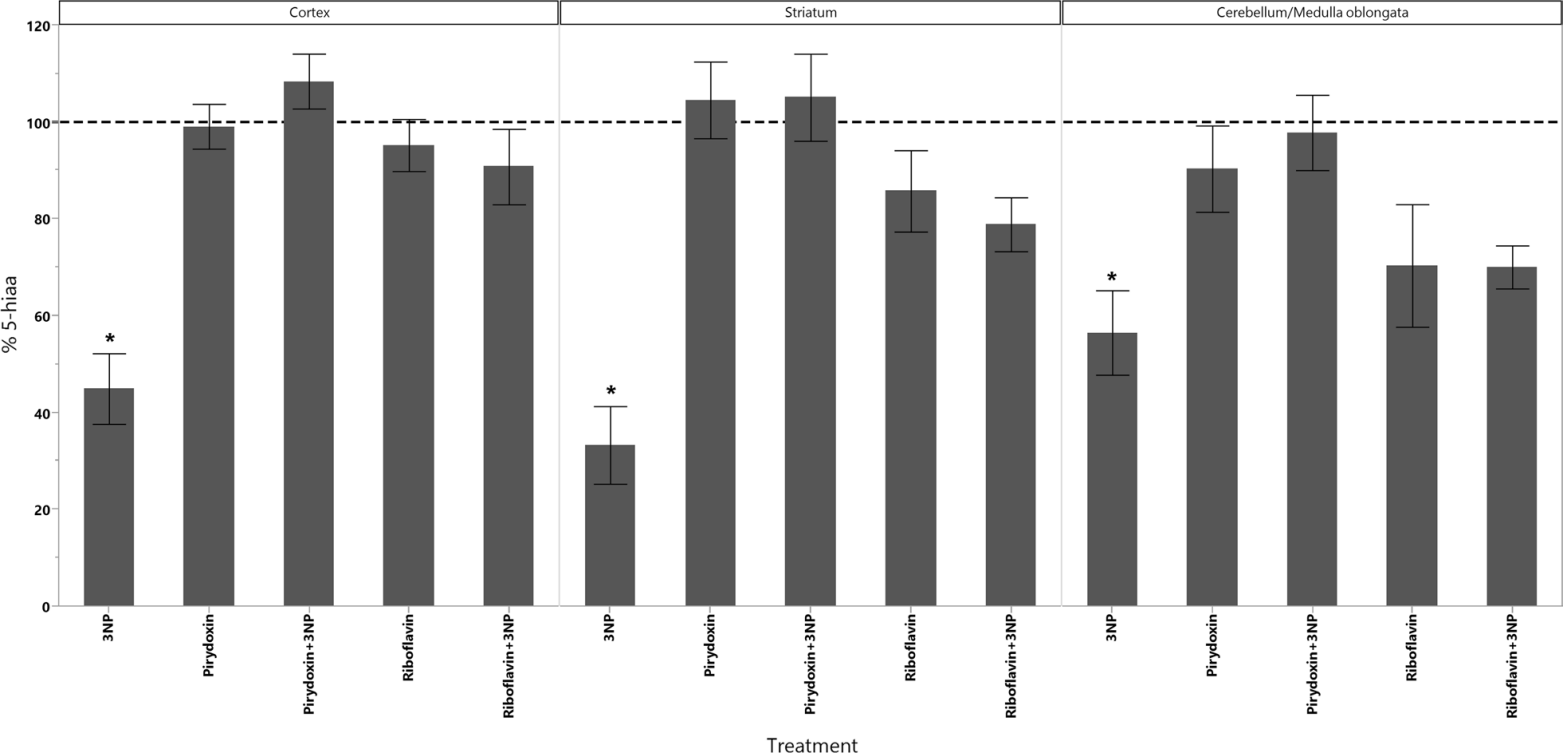
5-HIAA levels in brain of rats treated with NaCl (G1), 3-Nitropropionic acid 3-NPA (G2), Riboflavin B_2_ (G3), Riboflavin B_2_ + 3-NPA (G4), Pyridoxine B_6_ (G5) and Pyridoxine B_6_ + 3-NPA (G6). Data presented as Mean ± SD values of percentage with respect to NaCl control group. Assays were made by triplicate. Cortex: Anova F = 11.06; *p* < 0.0001; *p < 0.0004 3-NPA versus control, B_2_, B_2_ + 3-NPA, B_6_, B_6_ + 3-NPA. Striatum: Anova F = 10.3; *p* < 0.0001; *p < 0.006 3-NPA versus control, B_2_, B_2_ + 3-NPA, B_6_, B_6_ + 3-NPA. Cerebellum/medulla oblongata: Anova F = 4.9; *p* = 0.002; *p < 0.02 3-NPA versus control and B_6_ + 3-NPA

**Fig. 2:**
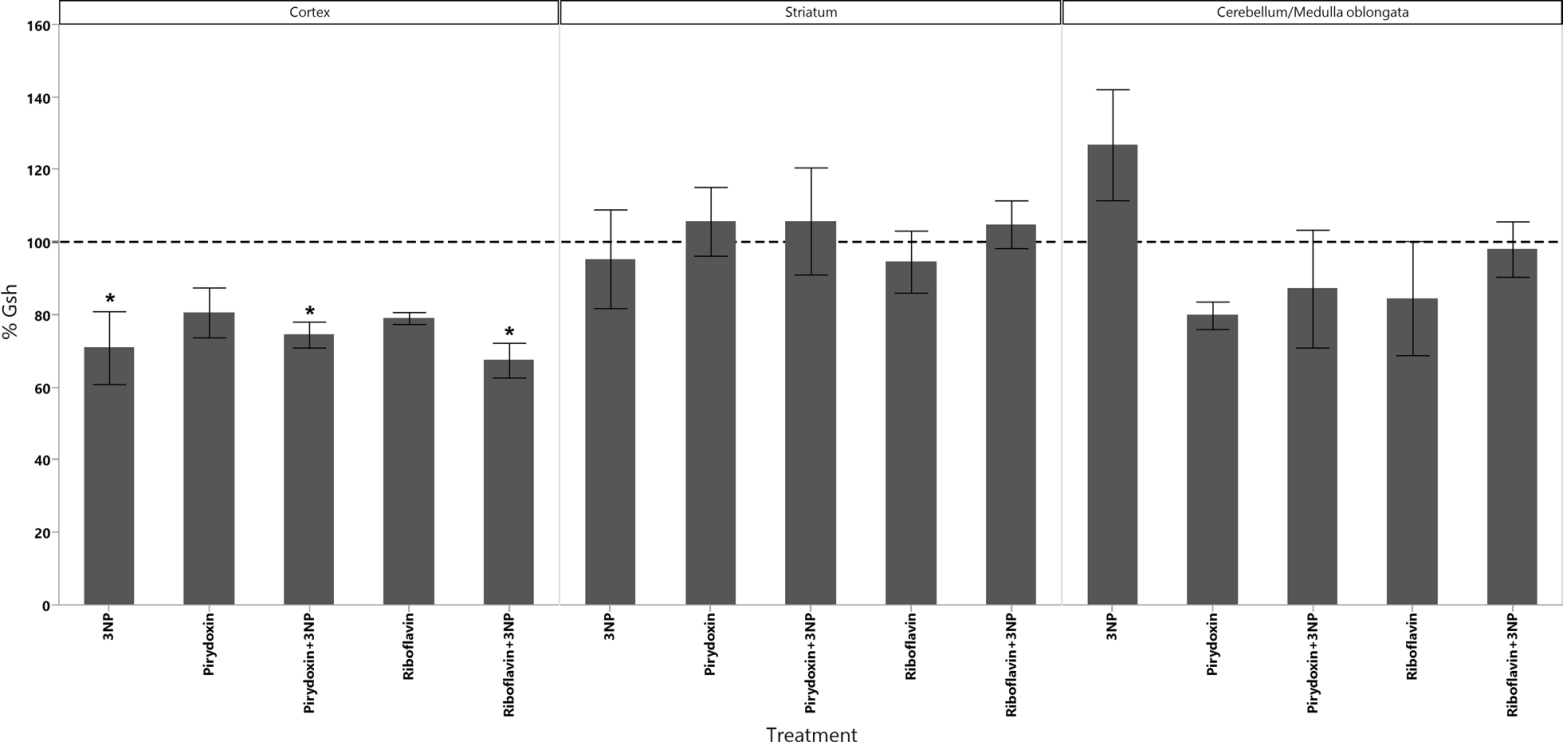
GSH levels in brain of rats treated with NaCl (G1), 3-Nitropropionic acid 3-NPA (G2), Riboflavin B_2_ (G3), Riboflavin B_2_ + 3-NPA (G4), Pyridoxine B_6_ (G5) and Pyridoxine B_6_ + 3-NPA (G6). Data presented as Mean ± SD values of percentage with respect to NaCl control group. Assays were made by triplicate. Cortex: Anova F = 3.97; *p* = 0.008; *p < 0.05 control versus 3-NPA, B_2_ + 3-NPA, B_6_ + 3-NPA. Striatum: Anova F = 0.02; *p* = 0.95. Cerebellum/medulla oblongata: Anova F = 1.8; *p* < 0.13

**Fig. 3:**
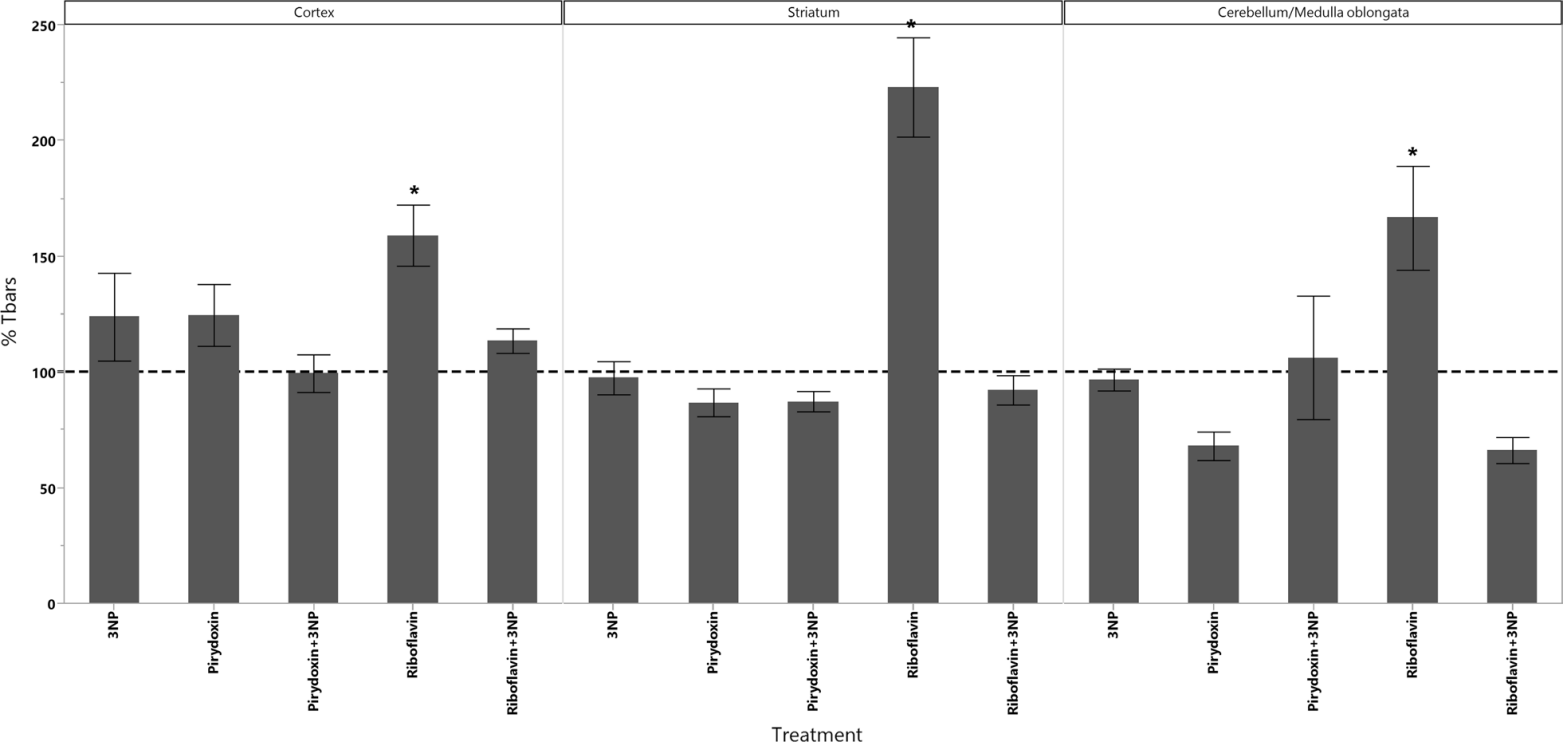
TBARS levels in brain of rats treated with NaCl (G1), 3-Nitropropionic acid 3-NPA (G2), Riboflavin B_2_ (G3), Riboflavin B_2_ + 3-NPA (G4), Pyridoxine B_6_ (G5) and Pyridoxine B_6_ + 3-NPA (G6). Data presented as Mean ± SD values of percentage with respect to NaCl control group. Assays were made by triplicate. Cortex: Anova F = 4.60; *p* = 0.003; *p < 0.05 B_2_ versus control, B_2_ + 3-NPA, B_6_ + 3-NPA. Striatum: Anova F = 21.5; *p* < 0.0001; *p < 0.0001 B_2_ versus control, 3-NPA, B_2_ + 3-NPA, B_6_, B_6_ + 3-NPA. Cerebellum/medulla oblongata: Anova F = 5.21; *p* = 0.002; *p < 0.05 B2 versus control, 3-NPA, B_2_ + 3-NPA, B_6_

**Fig. 4:**
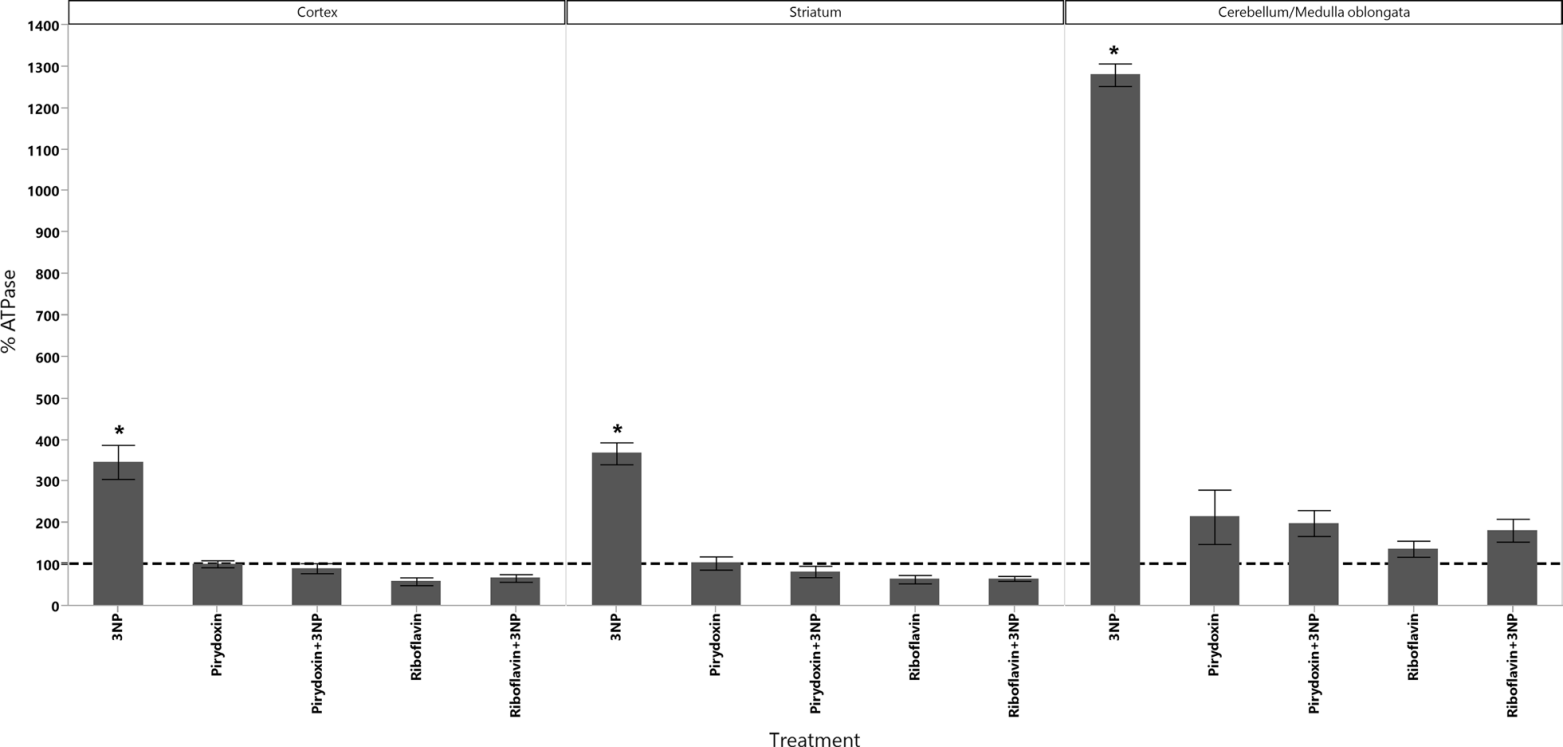
Ca^2+^, Mg^2+^ATPase activity in brain of rats treated with NaCl (G1), 3-Nitropropionic acid 3-NPA (G2), Riboflavin B_2_ (G3), Riboflavin B_2_ + 3-NPA (G4), Pyridoxine B_6_ (G5) and Pyridoxine B_6_ + 3-NPA (G6). Data presented as Mean ± SD values of percentage with respect to NaCl control group. Assays were made by triplicate. Cortex: Anova F = 36.8; *p* < 0.0001; *p < 0.001 3-NPA versus control, B_2_, B_2_ + 3-NPA, B_6_, B_6_ + 3-NPA. Striatum: Anova F = 65.01; *p* < 0.0001; *p < 0.0001 3-NPA versus control, B_2_, B_2_ + 3-NPA, B_6_, B_6_ + 3-NPA. Cerebellum/medulla oblongata: Anova F = 134.3; *p* < 0.0001; *p < 0.0001 3-NPA versus control, B_2_, B_2_ + 3-NPA, B_6_, B_6_ + 3-NPA

**Fig. 5:**
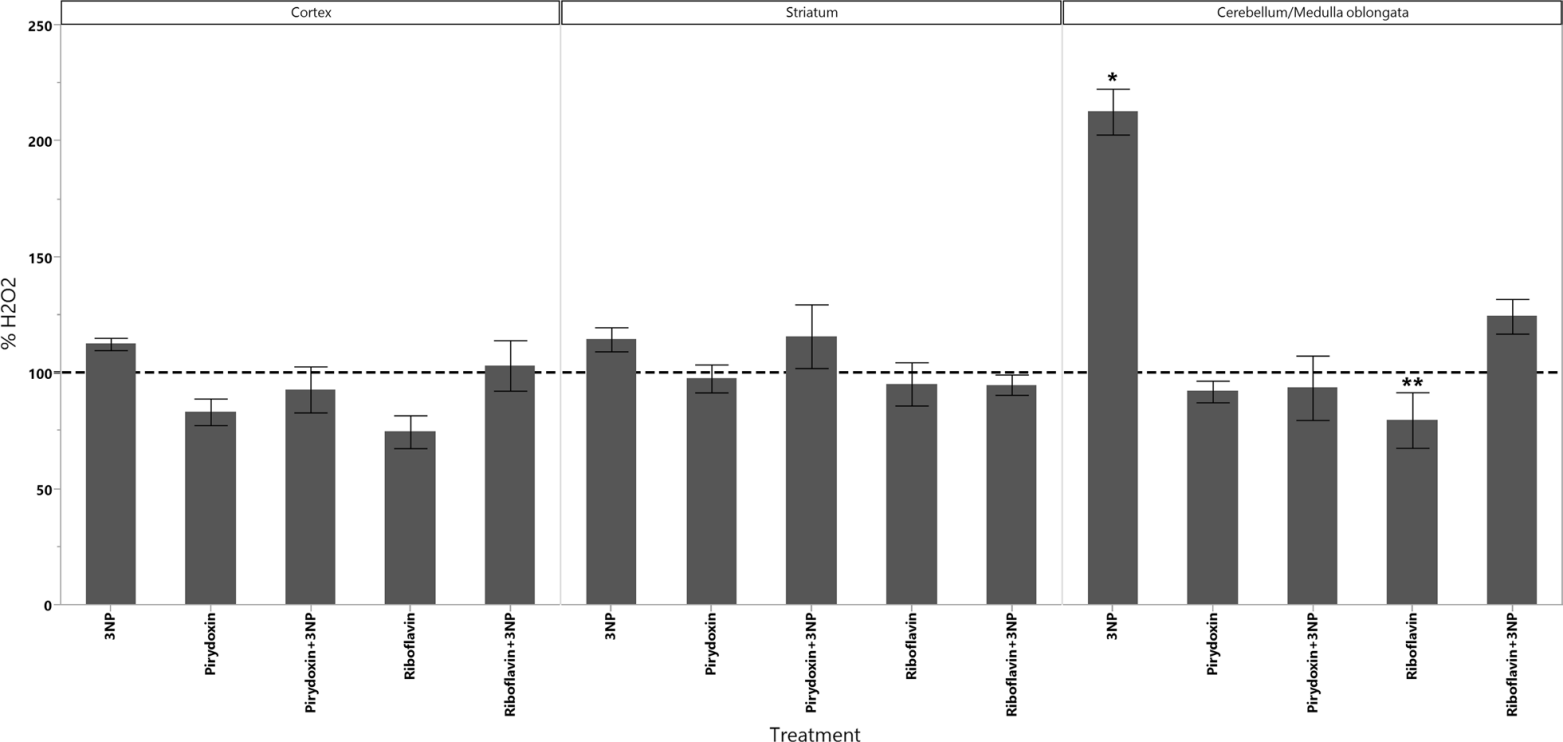
H_2_O_2_ levels in brain of rats treated with NaCl (G1), 3-Nitropropionic acid 3-NPA (G2), Riboflavin B_2_ (G3), Riboflavin B_2_ + 3-NPA (G4), Pyridoxine B_6_ (G5) and Pyridoxine B_6_ + 3-NPA (G6). Data presented as Mean ± SD values of percentage with respect to NaCl control group. Assays were made by triplicate. Cortex: Anova F = 2.15; *p* = 0.08. Striatum: Kruskal–Wallis X^2^ = 4.4; *p* = 0.4. Cerebellum/medulla oblongata: Anova F = 19.9; *p* < 0.0001; *p < 0.0001 3-NPA versus control, B_2_, B_2_ + 3-NPA, B_6_, B_6_ + 3-NPA. **p = 0.03 B_2_ versus B_2_ + 3-NPA

In cortex, the administration of 3-NPA produced a significant increase in the levels of dopamine as well as a significant decrease in the levels of 5-HIAA (Fig. 1). GSH decreased in all animals groups that received the treatments however, the decrease of GSH had statistical significant difference only in those treated with Rivoflavin (B_2_), Rivoflavin (B_2_) + 3-NPA and Pyridoxine (B_6_) + 3-NPA when compared with the control group (Fig. 2). Significant lipid peroxidation was appreciated in animals treated with Rivoflavin in comparison with control, B_2_ + 3-NPA and B_6_ + 3-NPA groups (Fig. 3). Calcium Magnesium dependent ATPase activity increased significantly in the group of animals that received 3-NPA when compared with other treatments (Fig. 4). In this region, the changes in H_2_O_2_ levels did not have statistical differences among treated and control groups (Fig. 5).

In striatum similar results as those observed in cortex were found. 3-NPA decreased 5-HIAA concentration (Fig. 1). Rivoflavin administration produced an increase in lipid peroxidation and 3-NPA treated animals showed significant increase in ATPase activity (Fig. 4).

**Fig. 6:**
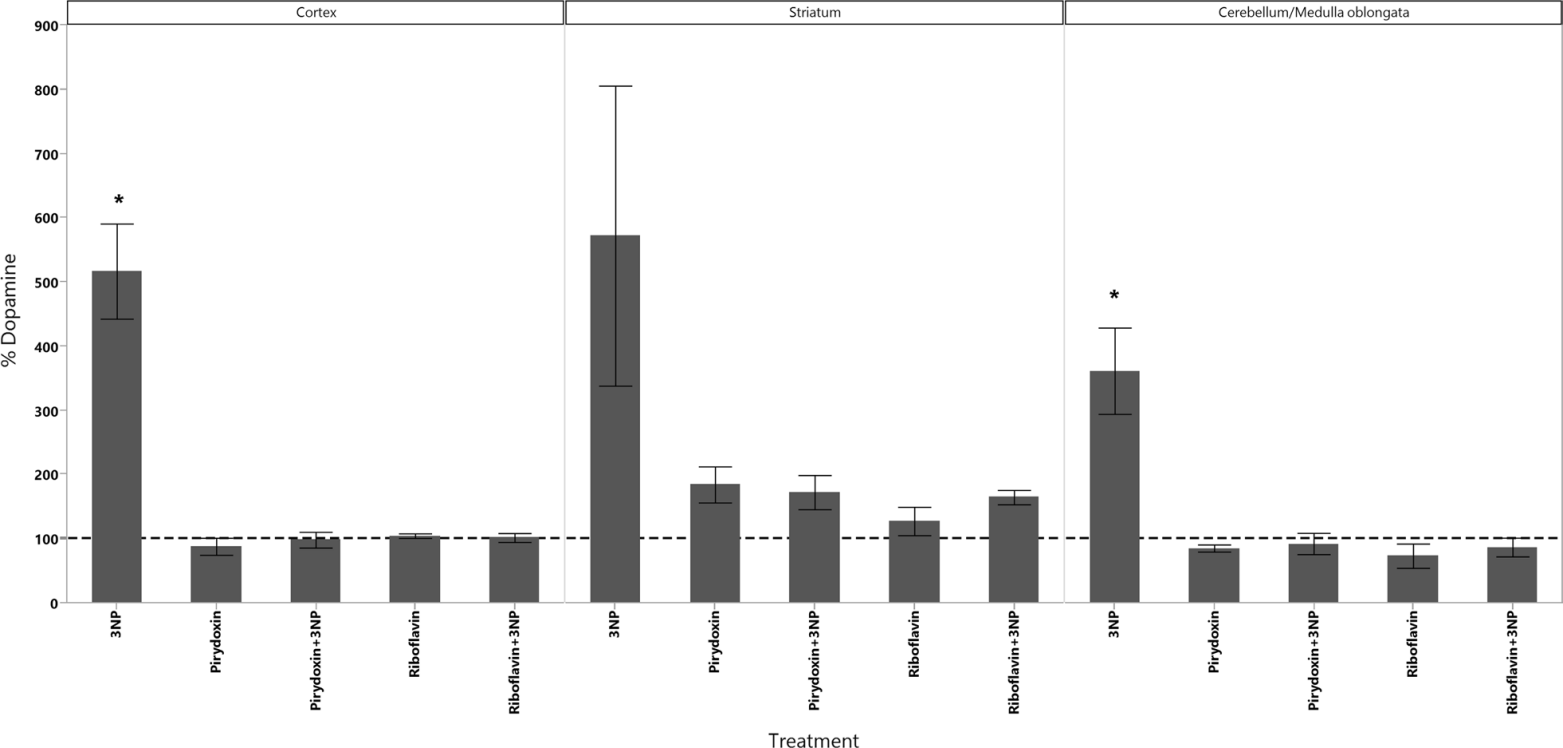
Dopamine levels in brain of rats treated with NaCl (G1), 3-Nitropropionic acid 3-NPA (G2), Riboflavin B_2_ (G3), Riboflavin B_2_ + 3-NPA (G4), Pyridoxine B_6_ (G5) and Pyridoxine B_6_ + 3-NPA (G6). Data presented as Mean ± SD values of percentage with respect to NaCl control group. Assays were made by triplicate. Cortex: Anova F = 41.3; *p* < 0.0001; *p < 0.0001 3-NPA versus control, B_2_, B_2_ + 3-NPA, B_6_, B_6_ + 3-NPA. Striatum: Kruskal–Wallis X^2^ = 11.6; *p* = 0.04 (No comparisons were made due 3-NPA data dispersion). Cerebellum/medulla oblongata: Anova F = 18.1; *p* < 0.0001 3-NPA versus control, B_2_, B_2_ + 3-NPA, B_6_, B_6_ + 3-NPA

In the cerebellum/Medulla oblongata of the animals that received 3-NPA alone, Dopamine, H_2_O_2_ and ATPase activity increased while 5-HIAA levels were found to. In this region significant lipid peroxidation increment was observed in Rivoflavin treated group when compared with the rest of the treated animals decrease (Figs. 1, 4, 5, 6).

## Discussion

Evidence has shown that Huntington’s disease (HD) can be caused by mitochondrial toxin produced as a result of striatal damaged that is provoked when the transmitter dopamine is metabolized by monoamine oxidase enzymes [4]. Also, HD is a destructive neurodegenerative disorder and indicates dysfunction in the neurons that may eventually lead to death of selected brain regions; especially, the striatum and cerebral cortex [5]. In recent studies, the dendritic spine density of striatal projection neurons was reported to be seriously decreased after 3-nitropropionic acid treatment [23]. This finding is in conformity with the results of the present study where dopamine levels increased in cortex, striatum and cerebellum/medulla oblongata regions of animals that received 3-nitropropionic acid treatment. Moreover, it is possible that the increase in dopamine turnover produces an increase in oxygen radical by monoamine oxidase activity.

H_2_O_2_ concentration increased in cerebellum/medulla oblongata regions in animals treated with 3-NPA. This demonstrates that reactive oxygen species is the primary event in 3-NPA toxicity. In the same brain regions, lipid peroxidation increased in animals treated with riboflavin. Such increase may be due to the association of this substance with increased mitochondrial energy metabolism that is probably responsible for the high rate of oxidative metabolic activity in the brain which gives rise to intense production of reactive oxygen metabolite and subsequently to the generation of free radicals implicated in the pathogenesis of neurological disorders. These results may have relation with the reports of Kumar et al. [24] and Kaur et al. [25], which suggest that 3-NPA depleted GSH in cortex, although the present study was made with young animal models.

Calcium and magnesium-dependent ATPase activity increased in cortex, striatum and cerebellum/medulla oblongata regions of the animals that received 3-NPA alone. This could be attributed to changes in the affinity of the enzyme [26]. These results may have relation with the reports of Naziroğlu et al. [27], which suggest that increase in Ca^2+^-ATPase activities may have protective effects against substances that induce brain injury by inhibiting free radical production, regulating calcium-dependent processes and supporting the antioxidant redox system.

## Conclusion

The results of the present study suggest that the partial increase in antioxidant capacity in brain due to B_2_ and B_6_ vitamins promotes an effect in dopamine or serotonin metabolisms when we consider 3-NPA capacity to generate oxidative stress.

We recommend that further studies be carried out to investigate the possible relationship between B_2_ and B_6_ vitamins on dopamine and serotonin levels in different animal models. Undoubtedly, we believe that this would help in the study of neurodegenerative diseases.

## Abbreviations

AO: antioxidant
CNS: central nervous system
DA: dopamine
DNA: deoxyribonucleic acid
EAA: excitatory amino acids
FR: free radicals
GSH: glutathione
HD: Huntington’s disease
5-HIAA: 5-hydroxyindole-acetic acid
NOGSH: nitroso-glutathione
3-NPA: 3-nitropropionic acid
l-DOPA: l-3,4-dihydroxyphenylalanine
OPT: ortho-phthaldehyde
RNS: reactive nitrogen species
ROS: reactive oxygen species
Tbars.: thiobarbituric acid reactive substances

## References

[1] Rami A, Ferger D, Krieglstein J (1997). Blockade of calpain proteolytic activity rescues neurons from glutamate excitotoxicity. Neurosci Res.

[2] Aliev G, Obrenovich ME, Tabrez S, Jabir NR, Reddy VP, Li Y (2013). Link between cancer and Alzheimer disease via oxidative stress induced by nitric oxide-dependent mitochondrial DNA overproliferation and deletion. Oxid Med Cell Longev.

[3] Szabó A, Papp A, Nagymajtényi L (2005). Effects of 3-nitropropionic acid in rats: general toxicity and functional neurotoxicity. Arh Hig Rada Toksikol.

[4] Smith RR, Dimayuga ER, Keller JN, Maragos WF (2005). Enhanced toxicity to the catecholamine tyramine in polyglutamine transfected SH-SY5Y cells. Neurochem Res.

[5] Browne SE, Beal MF (2006). Oxidative damage in Huntington’s disease pathogenesis. Antioxid Redox Signal.

[6] Ashoori M, Saedisomeolia A (2014). Riboflavin (vitamin B2) and oxidative stress: a review. Br J Nutr.

[7] Calderón-Guzmán D, Hernández-Islas JL, Espitia-Vázquez I, Barragán-Mejía G, Hernández-García E, Santamaría-del Angel D (2004). Pyridoxine, regardless of serotonin levels, increases production of 5-hydroxytryptophan in rat brain. Arch Med Res.

[8] Tupe RS, Agte VV (2010). Effect of water soluble vitamins on Zn transport of Caco-2 cells and their implications under oxidative stress conditions. Eur J Nutr.

[9] Hogg N, Singh RJ, Kalyanaraman B (1996). The role of glutathione in the transport and catabolism of nitric oxide. FEBS Lett.

[10] Beckman JS, Beckman TW, Chen J, Marshall PA, Freeman BA (1990). Apparent hydroxyl radical production by peroxynitrite: implications for endothelial injury from nitric oxide and superoxides. Proc Natl Acad Sci USA.

[11] Gutteridge JM, Halliwell B (1990). The measurement and mechanism of lipid peroxidation in biological systems. Trends Biochem Sci.

[12] Vogt MC, Brüning JC (2013). CNS insulin signaling in the control of energy homeostasis and glucose metabolism: from embryo to old age. Trends Endocrinol Metab..

[13] Swapna I, Sathya KV, Murthy CR, Senthilkumaran B (2005). Membrane alterations and fluidity changes in cerebral cortex during ammonia intoxication. NeuroToxicol.

[14] Stefanello FM, Chiarani F, Kurek AG (2005). Methionine alters Na^+^, K^+^ ATPase activity, lipid peroxidation and nonenzymatic antioxidant defenses in rat hippocampus. Int J Dev Neurosci.

[15] Calderon GD, Juarez OH, Hernandez GE, Labra RN, Barragan MG, Trujillo JF (2013). Effect of an antiviral and vitamins A, C, D on dopamine and some oxidative stress markers in rat brain exposed to ozone. Arch Biol Sci Belgrade.

[16] Calderón GD, Osnaya BN, García AR, Hernández GE, Guillé PA (2008). Levels of glutathione and some biogenic amines in the human brain putamen after traumatic death. Proc West Pharmacol Soc.

[17] Beck O, Palmskog G, Hultman E (1977). Quantitative determination of 5-hydroxyindole-3-acetic acid in body fluids by HPLC. Clin Chim Acta.

[18] Hissin PJ, Hilf R (1974). A flurometric method for determination of oxidized and reduced glutathione in tissue. Anal Biochem.

[19] Calderón-Guzmán D, Espitia-Vázquez I, López-Domínguez A, Hernández-García E, Huerta-Gertrudis B, Juárez-Olguín H (2005). Effect of toluene and nutritional status on serotonin, lipid peroxidation levels and Na^+^/K^+^ATPase in adult rat brain. Neurochem Res.

[20] Fiske CH, Subbarow Y (1925). The colorimetric determination of phosphorus. J Biol Chem.

[21] Asru KS (1972). Colorimetric assay of catalase. Anal Biochem.

[22] Castilla-Serna L (2011). Practical statistic guide for human health.

[23] Mu S, Lin E, Liu B, Ma Y, OuYang L, Li Y, Chen S, Zhang J, Lei W (2014). Melatonin reduces projection neuronal injury induced by 3-nitropropionic acid in the rat striatum. Neurodegener Dis.

[24] Kumar P, Kalonia H, Kumar A (2010). Protective effect of sesamol against 3-nitropropionic acid-induced cognitive dysfunction and altered glutathione redox balance in rats. Basic Clin Pharmacol Toxicol.

[25] Kaur N, Jamwal S, Deshmukh R, Gauttam V, Kumar P (2015). Beneficial effect of rice bran extract against 3-nitropropionic acid induced experimental Huntington’s disease in rats. Toxicol Rep.

[26] Hoskins B, Ho IK, Meydrech EF (1985). Effects of aging and morphine administration on calmodulin and calmodulin-regulated enzymes in striata of mice. J Neurochem.

[27] Naziroğlu M, Kutluhan S, Yilmaz M (2008). Selenium and topiramate modulates brain microsomal oxidative stress values, Ca^2+^-ATPase activity, and EEG records in pentylentetrazol-induced seizures in rats. J Membr Biol.

